# Retrospective analysis of medical emergencies in an oral emergency department

**DOI:** 10.4317/medoral.25947

**Published:** 2023-04-26

**Authors:** Xiao Shao, Jie Bai, Ai-Ping Ji, Wei Sun

**Affiliations:** 1 Department of Oral Emergency,Peking University School and Hospital of Stomatology and National Center of Stomatology and National Clinical Rescarch Center for Oral Diseases and National Engincering Research Center of Oral Biomaterials and Digital Medical Devices and Beijing Key Laboratory of Digital Stomatology and Research Center of Engineering and Technology for computerized Dentistry Ministry of Health and NMPA Key Laboralory for Dental Materials,Beijing,China

## Abstract

**Background:**

To retrospectively analyze the rescue of medical emergencies and critical patients in the oral emergency department in a hospital during the past 14 years; analyze the general condition of patients, their diagnosis, etiological factors, and outcomes of the disease, so as to improve the ability of oral medical staff to deal with emergencies; and optimize the emergency procedures and resource allocation in such departments.

**Material and Methods:**

Data and related information of critical patient emergency rescue from the Emergency Department of the Hospital of Stomatology, Peking University from January 2006 to December 2019, were analyzed.

**Results:**

A total of 53 critical patients were rescued in the oral emergency department in the past 14 years, which is an average of four cases per year, with an incidence rate of 0.00506%. The main type of emergency included hemorrhagic shock and active hemorrhage, with the highest incidence being in the age group of 19-40 years old. Among these cases, 67.92% (36/53) developed emergency and critical diseases before visiting the oral emergency department and 41.51% (22/53) had systemic diseases. After rescue, a total of 48 patients (90.57%) had sTable vital signs and 5 (9.43%) died.

**Conclusions:**

Oral doctors and other medical staff should be able to rapidly identify medical emergencies in oral emergency departments and commence emergency treatment. The department should be equipped with relevant first-aid drugs and devices, and medical staff should be regularly trained in practical first-aid skills. Patients with oral and maxillofacial trauma, massive hemorrhage and systemic diseases should be evaluated and treated according to their conditions and systemic organ function to prevent and reduce medical emergencies.

** Key words:**Emergency treatment, medical emergency, oral emergency department.

## Introduction

Oral diagnosis and treatment involve invasive procedures and involve long treatment time. Patients with oral diseases are prone to be nervous and anxious, and their health deteriorates with age (1,2). In addition, medical emergencies may occur during diagnosis and treatment. Previous reports on medical emergencies in the stomatology department were mostly derived from foreign literature, where the incidence of medical emergencies was 0.029% (3-5), and 19%-57% of stomatologists experienced at least one medical emergency in oral diagnosis and treatment in the past one year (4,6). However, such studies are mainly conducted retrospectively through questionnaire survey, which may produce information biases, and the handling and outcome of medical emergencies in the stomatology department are not reported. In this paper, data were obtained from the critical patient rescue record reporting platform and electronic medical record database of the hospital. Cases with medical emergencies in the Emergency Department of the Hospital of Stomatology during the past 14 years were also retrospectively analyzed, to summarize the characteristics of their occurrence and develop key points of treatment. The goal is to improve the ability of oral medical staff to cope with oral medical emergencies, and optimize first-aid procedures and resource allocation in stomatology departments.

## Material and Methods

Data of all patients who were rescued in the Emergency Department of Peking University Hospital of Stomatology from January 2006 to December 2019 were collected from the critical patient rescue record reporting platform and electronic medical record database of the hospital, including patients’ general conditions (age, gender, and general condition), disease diagnosis, whether oral invasive procedures were performed and the types of procedures, types of medical emergencies, causes, and emergency handling and outcome.

A total of 53 critical patients were rescued in the emergency department of the hospital during the past 14 years, which is an average of four cases per year, with an incidence rate of 0.00506%. Among them, there were 34 males and 19 females, with a ratio of 1.79:1; the youngest patient was 8 years old and the oldest was 86 years old, with a median age of 43; 92.5% (49/53) of patients were visiting the oral emergency department, 5.7% (3/53) were family members of patients and waiting patients, and 1.8% (1/53) were passers-by near the hospital.

Data were sorted out and analyzed using SPSS 19.0 software.

## Results

- Types of medical emergencies and age distribution

Among all the 53 critical patients who were rescued, the primary types of medical emergencies included hemorrhagic shock and active hemorrhage (Table 1), which were caused by maxillofacial traumatic hemorrhage (64.7%, 11/17), tooth extraction and postoperative hemorrhage (29.4%, 5/17), and mass rupture hemorrhage (1/17). There was no statistical difference in the incidence of various medical emergencies (Kruskal Wallis test, χ2 = 12, P = 0.446).

**Table 1:**
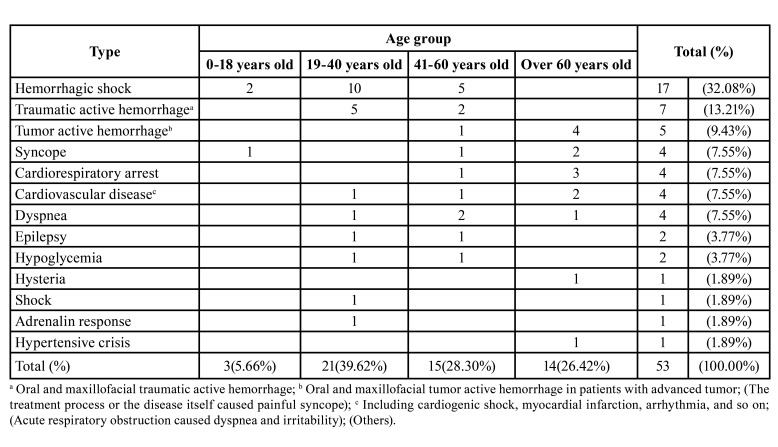
Type and age distribution of medical emergencies in the emergency department of a specialized stomatological hospital.

Patients in the age group of 19-40 years old had the highest incidence of trauma. Hemorrhagic shock and traumatic active hemorrhage mainly occurred in young and middle-aged patients; oral and maxillofacial tumor active hemorrhage, syncope, cardiac arrest, cardiovascular disease, and dyspnea were common among patients over 40 years old (Table 1).

- Relationship between medical emergencies and oral treatment

Among the 53 critical patients who were rescued, 67.9% developed emergency and critical conditions before visiting the oral emergency department, including shock, hemorrhage, and dyspnea; 15.2% suffered from an emergency after emergency treatment, including syncope, shock, adrenalin response, and so on; only a small number suffered from an emergency during oral emergency treatment, including shock, syncope, and hypoglycemic reaction (Table 2).

- Systemic diseases of patients with medical emergencies

Among the 53 patients, 41.51% (22/53) were suffering from systemic diseases, including coronary heart disease, malignancy, diabetes mellitus, hypertension, and so on; patients with cardiorespiratory arrest and who died were suffering from severe systemic diseases.

- First-aid methods during medical emergencies

The vital signs and symptomatic treatment of oxygen uptake and fluid infusion were monitored for most of the patients. Local treatment included hemostasis at the position of hemorrhage, suturing, anti-hemorrhagic shock, and so on, and there was one case of emergency tracheotomy for airway obstruction and mechanical assistance, and preliminary treatment of CPR early warning process was started (Table 3).

Immediately after the occurrence of medical emergencies, specialists in the oral emergency department received and examined the patients and determined whether senior oral and maxillofacial surgeons, clinical physicians, and anesthesiologists should be notified to assist in the rescue; the conditions of 45.28% (24/53) of patients could be controlled after treatment by specialists in the oral emergency department, and 54.72% (29/53) had to be rescued with the assistance of doctors from other departments.

**Table 2:**
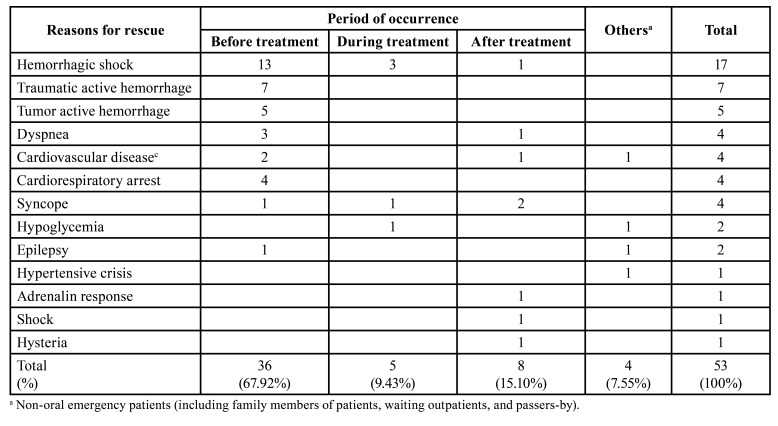
Relationship between emergency medical events and oral treatment in emergency department of stomatological hospital.

**Table 3:**
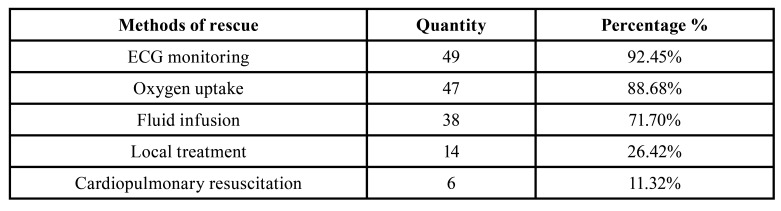
The first aid method of the emergency department of stomatological hospital.

- First-aid outcome of patients

Ten patients (18.87%) left the hospital on their own with sTable conditions after rescue; 13 (24.53%) were admitted to the oral and maxillofacial wards of the Hospital of Stomatology for further treatment; 25 (47.17%) were transferred to the general hospital for further treatment after their vital signs were stabilized; 5 (9.43%) died.

## Discussion

Complications and some unpredicTable special cases that occur during oral diagnosis and treatment, which endanger the life and health of patients, patients’ companions, and medical staff, are referred to as oral medical emergencies. The oral emergency department mainly receives patients with oral and maxillofacial trauma, hemorrhage, infection, and acute pain (7), and common treatments include debridement and suturing, drainage, opening of pulp chamber, analgesia, and other measures under local anesthesia. However, there are still critical patients, and oral medical emergencies are common even in oral departments or clinics (2,8-11).

A total of 1.047 million patients were received in the Emergency Department of the Hospital of Stomatology in the past 14 years, with an incidence rate of oral medical emergencies of 0.00506%, lower than that reported abroad (3,12,13). In foreign literature, syncope is the most prevalent oral medical emergency (2,6,14), and accounts for over 50% of all medical emergencies in some reports; yet, in the medical emergencies in the Emergency Department of the Hospital of Stomatology, syncope accounted for only 7.55%, while hemorrhagic shock had a higher proportion. The reasons for the same are analyzed as follows:

First, the patients were from different sources: oral medical emergencies reported in foreign literature occurred in stomatology clinics, while the patients in this study were from the oral emergency department, and many patients with maxillofacial trauma preferred to visit the oral emergency department.

Second, patients with syncope and hemorrhagic shock had similar symptoms in the early stage and were indistinguishable, and foreign literature mainly utilized questionnaires for oral practitioners and did not consider medical records, which may lead to information biases.

Third, patients remained in the supine position during the diagnosis and treatment in the oral emergency department, which could maintain sufficient blood supply to the brain and avoid the occurrence of transient syncope, such as postural hypotension. For operations that may cause pain, such as opening of pulp chamber, abscess incision drainage, and curettage of teeth extraction socket, effective local anesthesia should be taken in advance for analgesia, and an empty stomach should be avoided before local anesthesia; patients’ systemic conditions should be evaluated before invasive treatment; attention should be paid to patients’ response to oral treatment during operation, while soothing patients to reduce their tension and minimizing the adverse stimulation to patients during operation, so as to reduce the incidence of medical emergencies.

In this study, the incidence of medical emergencies in the oral emergency department was the highest among the young and middle-aged patients, and were mainly caused by hemorrhagic shock and traumatic active hemorrhage. Oral and maxillofacial trauma occurred frequently among young and middle-aged patients; symptoms such as craniocerebral injury, injury of vital organs, and massive hemorrhage caused by trauma may cause syncope, shock, and even the failure of vital organs before treatment; and fractures may cause glossocoma, leading to respiratory obstruction and other symptoms (3,15,16). Therefore, for patients with maxillofacial trauma, the cause of injury and the symptoms should be enquired in detail before treatment. For patients who suffered blunt force trauma to the head and face, fell from a height or were injured in other situations, and developed headache, nausea and vomiting, transient loss of consciousness (TLOC), weak consciousness or other symptoms during and after the trauma, the brain and vital organs should be examined immediately after simple and effective local hemostasis, and the respiratory tract examined for obstructions, to rule out potential traumatic craniocerebral injury and life-threatening conditions. Changes in vital signs should be closely monitored during the treatment of oral and maxillofacial trauma (5,16). In implementing first-aid measures, relevant items, such as ECG monitors, oxygen uptake apparatuses, vacuum extractors, laryngoscopes, trachea cannulas, tracheotomy sets, simple respirators, and first-aid medicines, should be prepared (4,17). During rescue, venous channels should be quickly established to clear foreign matters in the respiratory tract and keep the airway unobstructed (18).

In this study, 41.51% of the patients had systemic diseases, and those with cardiorespiratory arrest and who died, suffered from severe systemic diseases. In cases related to respiratory, circulatory, and other systemic diseases, the number of middle-aged and elderly patients over 40 years old was three times as many as that in any other age group. Therefore, the systemic conditions and past medical history of patients requiring local anesthesia and invasive treatment, especially the middle-aged and elderly patients, should be enquired in detail and evaluated comprehensively according to their conditions and systemic organ function, to avoid and reduce medical emergencies caused by systemic diseases triggered or aggravated by oral irritation (13,16).

In this study, 67.9% of the patients developed emergency and critical conditions before visiting the oral emergency department, such as shock, hemorrhage, dyspnea, cardiovascular disease, and even cardiorespiratory arrest. Doctors in the stomatology department, especially in the oral emergency department, should be able to rapidly identify emergency and critical conditions and start primary treatment (1,2,4), which is the key to minimizing the incidence and mortality rate. In addition to identifying and managing common oral medical emergencies, stomatology departments should also be provided with relevant first-aid medicine and equipment, and regular training in practical first-aid skills, including medium/advanced life support measures, should be provided to medical staff.

Cardiorespiratory arrest is a rare emergency reported in Chinese and foreign literature with respect to oral medical emergencies, however, it may lead to serious consequences if it is not promptly and effectively treated. Therefore it is vital to include emergency training for cardiorespiratory arrest (3,6,19). In this study, there was one case of cardiorespiratory arrest in the oral emergency department every three and a half years, and the proportion was higher than the results of questionnaire surveys conducted in foreign dental clinics, whereas, no such incident was reported in domestic outpatient surveys. Cardiac arrest refers to the sudden stop of cardiac output caused by ventricular arrest due to various reasons. Cardiopulmonary resuscitation (CPR) refers to a series of emergency measures, where artificial methods are used to establish and restore circulatory and respiratory functions, actively protect the brain, and completely restore its function in patients with cardiac and respiratory arrest (16,20). Studies have shown that the longer the interval between cardiac arrest and CPR, the longer it is for return of spontaneous circulation (ROSC), and the clinical prognosis of patients receiving pre-hospital emergency treatment is poorer. Therefore in case of cardiovascular emergencies, especially cardiac arrest, CPR should be provided immediately after accurately determining the breathing, pulse, and consciousness state of the patient. ECG monitoring equipment should also be routinely made available in the oral emergency and stomatology departments. Doctors in the oral emergency department should master the knowledge of interpreting ECG results, and nursing staff should be able to connect the ECG monitor skillfully and correctly. Studies have shown that tooth extraction for patients with cardiovascular disease under ECG monitoring can significantly improve safety of the procedure, and thus stomatology departments should consider ECG monitoring in the oral treatment of patients with cardiovascular disease (21).

In this study, most of the patients with medical emergencies were monitored for vital signs and treated with oxygen uptake and fluid infusion, and more than half of the patients were rescued with the assistance of oral and maxillofacial surgeons, clinical physicians, and anesthesiologists; only 26.4% of the patients required local treatment, including hemostasis at the position of hemorrhage and suturing, and emergency tracheotomy. More than 70% of the patients had to be transferred to oral and maxillofacial wards or the general hospital for further treatment after their vital signs stabilized. In case of complex systemic diseases, oral doctors may not be able to resolve the emergency on their own, but they should be able to identify the oral medical emergencies, correctly evaluate patients’ physiological status, adopt monitoring of vital signs and basic life support in time, and start emergency and critical condition rescue procedures and consultations, to effectively mitigate the impact of the medical emergency. The General Dental Council, UK (19) has highlighted that it is the responsibility of all dentists to undergo training to cope with possible medical emergencies in dental practice; dental hygienists should attend a basic life support training course every two years to ensure that they can be proficient in CPR skills and learn about the latest CPR guidelines in time (3,6,19).

## Conclusions

Despite the low incidence of medical oral emergencies, they may lead to serious consequences. In addition to professional diagnosis and treatment, stomatology hospitals should consistently improve their emergency response to clinical medical emergencies. Such incidents are frequent in oral emergency departments, but may also inevitably occur during daily outpatient consulting. Medical staff should learn about the types of possible medical emergencies in the stomatology department and relevant emergency measures, participate regularly in first-aid drills, and be able to identify medical emergencies and provide first-aid immediately in case of an emergency. Besides, stomatology related departments, general hospitals should establish a necessary green channel consultation mechanism in collaboration with the oral and maxillofacial surgical department, clinical internal medicine department, emergency department, ICU, and anesthesiology department, to coordinate and provide higher-level rescue measures and life support within the shortest time after the occurrence of medical emergencies. Finally, an awareness program should be implemented to train oral medical staff to identify triggers and precursors to medical emergencies, to avoid the occurrence of unnecessary emergencies and mitigate the consequences.
